# Usefulness of Whole-Body Fluorine-18-Fluorodeoxyglucose Positron Emission Tomography in Patients with Neurofibromatosis Type 1: A Systematic Review

**DOI:** 10.1155/2012/431029

**Published:** 2012-09-09

**Authors:** Giorgio Treglia, Silvia Taralli, Francesco Bertagna, Marco Salsano, Barbara Muoio, Pierluigi Novellis, Maria Letizia Vita, Fabio Maggi, Alessandro Giordano

**Affiliations:** ^1^Institute of Nuclear Medicine, Catholic University of the Sacred Heart, 00168 Rome, Italy; ^2^Department of Nuclear Medicine, University of Brescia, 25123 Brescia, Italy; ^3^Institute of Radiology, Catholic University of the Sacred Heart, 00168 Rome, Italy; ^4^School of Medicine, Catholic University of the Sacred Heart, 00168 Rome, Italy; ^5^Department of Thoracic Surgery, Catholic University of the Sacred Heart, 00168 Rome, Italy

## Abstract

*Aim*. To systematically review the role of positron emission tomography (PET) with fluorine-18-fluorodeoxyglucose (FDG) in patients with neurofibromatosis type 1 (NF1). *Methods*. A comprehensive literature search of published studies regarding FDG-PET and PET/CT in patients with NF1 was performed. No beginning date limit and language restriction were used; the search was updated until December 2011. Only those studies or subsets in studies including whole-body FDG-PET or PET/CT scans performed in patients with NF1 were included. *Results*. We identified 12 studies including 352 NF1 patients. Qualitative evaluation was performed in about half of the studies and semiquantitative analysis, mainly based on different values of SUV cutoff, in the others. Most of the studies evaluated the role of FDG-PET for differentiating benign from malignant peripheral nerve sheath tumors (MPNSTs). Malignant lesions were detected with a sensitivity ranging between 100% and 89%, but with lower specificity, ranging between 100% and 72%. Moreover, FDG-PET seems to be an important imaging modality for predicting the progression to MPNST and the outcome in patients with MPNST. Two studies evaluated the role of FDG-PET in pediatric patients with NF1. *Conclusions*. FDG-PET and PET/CT are useful methods to identify malignant change in neurogenic tumors in NF1 and to discriminate malignant from benign neurogenic lesions.

## 1. Introduction

Neurofibromatosis type 1 (NF1) is an autosomal dominant disease with an incidence of 1 in 2,500 to 1 in 3,000 subjects. Neurofibroma, a benign peripheral nerve sheath tumor, is the most common tumor in NF1 patients and may manifest as focal nodular, cutaneous or subcutaneous lesion, intraforaminal spinal nerve root tumor, or plexiform neurofibroma (PNF). Patients with NF1 have an increased risk of developing malignant peripheral nerve sheath tumors (MPNSTs) with a life-time risk of 8–12% [1–6]. MPNSTs usually arise from preexisting benign PNF, metastasize widely, and frequently have a poor prognosis. Therefore, differentiating between benign and malignant tumors in patients with NF1 has important prognostic and therapeutic implications, but can be difficult, especially in individuals who have multiple benign tumors. Optimal management is dependent on early and accurate histological grading and staging of the disease, but MPNSTs are often difficult to detect and may metastasize to many different sites. Pain, rapid increase in size of a neurofibroma, and the development of neurological deficit are clinical indicators of malignancy, but may also be features of benign PNF; therefore, clinical symptoms cannot reliably discriminate between benign and malignant lesions [1–6].

Magnetic resonance (MR) and computed tomography (CT) can be used to determine the site and extent of the PNF, but are not reliable in discriminating with high accuracy between benign PNF and those that have degenerated into MPNST [1–6]. Currently, histology remains the gold standard for identifying malignant transformation within a PNF. However, this requires complete excision, which is frequently not technically feasible, and if core biopsy is performed, the focus of malignant change, particularly within a large heterogeneous tumor, may be missed. Moreover, histopathology and tumor grading of MPNST are not strictly correlated with prognosis in these patients [1–6].

Positron emission tomography (PET) with the glucose analogue fluorine-18-fluorodeoxyglucose (FDG) is a functional imaging technique which allows the visualization and quantification of glucose metabolism and reflects the increase in metabolism in malignant tumors. Previous studies have suggested that FDG-PET successfully detects soft tissue sarcomas and metastases, can give an indication of the histological grade, and can be helpful in detecting tumor recurrence in patients with sarcomas and in differentiating benign from malignant tumors [7, 8]. Based on these findings, a noninvasive metabolic biopsy that would reliably differentiate MPNSTs from benign neurofibromas could play a major role in the management of patients with NF1. Furthermore, FDG-PET could be potentially useful for surveillance and management of glioma in NF1 patients.

Several studies have shown the potential role of whole-body FDG-PET and PET/CT in patients with NF1; however, a systematic review of published data in this field is lacking and this represents the purpose of our study.

## 2. Methods

### 2.1. Search Strategy

A comprehensive computer literature search of the PubMed/MEDLINE, Scopus, and Embase databases was conducted to find relevant published articles about the role of whole-body FDG-PET in patients with NF1. We used a search algorithm that was based on a combination of the terms: (a) “Neurofibromatosis” or “Neurofibroma” and (b) “Positron Emission Tomography” or “PET.” No beginning date limit and language restriction was used; the search was updated until December 2011. To expand our search, references of the retrieved articles were also screened for additional studies.

### 2.2. Study Selection

Studies or subsets in studies investigating the role of whole-body FDG-PET and PET/CT in patients with NF1 were eligible for inclusion. Review articles or editorials, case reports, and preclinical studies were excluded from this paper. Only those studies or subsets in studies including whole-body FDG-PET or PET/CT scans performed in patients with NF1 were included.

Two researchers (GT and ST) independently reviewed the titles and abstracts of the retrieved articles, applying the inclusion and exclusion criteria mentioned above. Articles were rejected if they were clearly ineligible. The same two researchers then independently reviewed the full-text version of the remaining articles to determine their eligibility for inclusion.

### 2.3. Data Abstraction

For each included study, information was collected concerning basic study (author names, journal, year of publication, country of origin), PET device used (PET or PET/CT), and patient characteristics (number of patients with NF1 or neurogenic tumors, sex and mean age, number of neurogenic tumor lesions evaluated). At last, the main findings of the articles included in this paper are reported in the results.

## 3. Results

### 3.1. Literature Search

**Figure 1:**
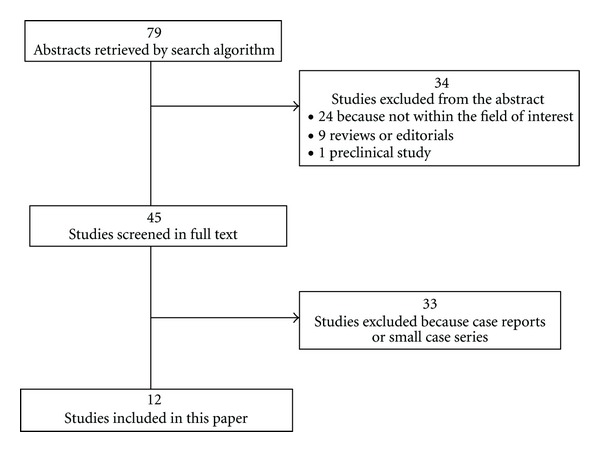


The comprehensive computer literature search from the PubMed/MEDLINE, Scopus, and Embase databases revealed 79 articles. Reviewing titles and abstracts, 24 articles were excluded because reported data were not within the field of interest of this paper; 9 articles were excluded as editorials or reviews; one article was excluded as preclinical study. Reviewing the full-text of the remaining articles, 33 articles were excluded as case reports. Lastly, 12 articles including 352 patients with NF1 were selected (Figure 1) [9–20]. These 12 studies were retrieved in full-text version; no additional studies were found screening the references of these articles. The characteristics of the included studies are presented in Tables 1 and 2.

### 3.2. Literature Data Report

#### 3.2.1. Role of FDG-PET and PET/CT in Differentiating Benign PNF from MPNST and in Detecting MPNST

In 2000, Ferner et al. [9] evaluated the ability of FDG-PET to detect malignant change of PNF in 18 patients with NF1. All PET scans were evaluated both qualitatively by visual inspection and semiquantitatively by calculation of standardized uptake value (SUV) in order to establish if the lesion was benign or malignant: those tumors with FDG uptake greater than the liver were assessed as malignant. Twenty-three PNFs were detected in 18 patients, and PET results were correlated with histological diagnosis or clinical followup that assessed 8 lesions as MPNSTs and 15 lesions as benign PNFs. Qualitative analysis of PET images interpreted 13 PNFs as benign and 10 as malignant: all the malignant tumors were identified, but two benign tumors were reported as malignant, with calculated values of sensitivity and specificity of 100% and 87%, respectively. The mean SUV was significantly higher in malignant tumors (mean 5.4 ± 2.4, range 2.7–8.4), than in benign tumors (mean 1.54 ± 0.7, range 0.56–3.3), and this difference was statistically significant (*P* = 0.002). However, there was an overlap between benign and malignant tumors in the SUV range 2.7–3.3. These findings suggested that FDG-PET is a useful noninvasive method to identify malignant change in PNF in patients with NF1. Furthermore, a cutoff of 2.5 for the SUV would allow to differentiate between benign and malignant lesions in most cases; nevertheless, an overlap between these two groups regarding SUV may be expected. The authors also suggested to calculate the SUV at about 200 minutes after FDG injection for an increased separation between benign and malignant lesions [9].

In 2003, Cardona et al. [10] assessed the diagnostic value and the therapeutic impact of FDG-PET in 13 patients with 25 neurogenic soft tissue tumors suspicious for MPNST. Fifteen tumors (60%) were detected in 5 patients with known NF1. FDG-PET was performed in all patients; qualitative and semiquantitative analysis (based on median SUV) was performed. Sensitivity and specificity were calculated for a SUV cutoff value of 1.8. FDG-PET results were correlated with histological reports and follow-up data that assessed 13 lesions as malignant and 12 lesions as benign. Visual analysis of suspected lesions by FDG-PET reported 9 tumors as benign and 16 as malignant; no malignant tumors were classified as benign, but 3 benign tumors were identified as malignant. MPNST (*n* = 13) showed a significantly 2.6-fold higher SUV (median 2.9; range: 1.8–12.3) compared to benign tumors (*n* = 12; median 1.1; range 0.5–1.8), and this difference was statistically significant (*P* < 0.001). At a SUV cut-off value of 1.8 measured 60 minutes after injection, FDG-PET distinguished between MPNSTs and benign neurogenic tumors with 100% of sensitivity and 83% of specificity. In addition, in 4 of 13 patients, FDG-PET provided additional information, which influenced the treatment plan. These findings suggested that FDG-PET allows discrimination of MPNST from benign neurogenic lesions, may improve preoperative tumor staging, and influences treatment, reducing the number of surgical procedures for benign lesions in NF1 patients [10].

In 2007, Bensaid et al. [13] investigated the usefulness of FDG-PET in detection of MPNST in 38 patients with NF1. Forty-nine suspected MPNSTs were included in the study. Analysis of PET images, based upon determination of tumor/liver binding ratio with a cutoff of 1.5 times hepatic binding, was used to classify lesions as nonsuspected or pathological. Histological analysis of suspected lesions or monitoring or excision of nonsuspected lesions was performed. In 8 patients, FDG-PET showed suspected lesions (12 tumors), and histological analysis revealed 6 MPNSTs. In 30 patients, PET scan showed nonsuspected lesions (37 tumors), and no malignant tumors were demonstrated either on histological examination or after a mean followup of 33.5 months. PET scan thus demonstrated a sensitivity and negative predictive value of 100%, specificity of 86%, and a positive predictive value of 50%. This study demonstrated the value of FDG-PET in detecting MPNST, even though false positive results require medical-surgical confirmation before any therapeutic decision [13].

In their retrospective study, Bredella et al. [14] assessed the role of PET and PET/CT using FDG in the detection of MPNST in 45 patients NF1. Twenty-seven patients underwent biopsy or surgical tumor resection and the remaining 18 patients were followed up clinically and with repeat imaging for a period of 1–5 years. Semiquantitative and qualitative evaluations of PET images were performed; maximum SUV was calculated and tumors with tracer uptake greater than the liver were classified as malignant, and those with uptake equal to or less than the liver were classified as benign. Fifty lesions were identified in 45 patients. Based on qualitative evaluation of the suspected lesions, 26 tumors were characterized as benign and 24 as malignant: there were 8 false positive results and one false negative finding on FDG-PET. SUV for MPNST ranged from 3.8 to 13.0 with a mean of 8.5 ± 0.63 on FDG-PET. Benign peripheral nerve sheath tumors showed SUV ranging from 0 to 5.3 with a mean of 1.5 ± 0.37 on FDG-PET. The difference between the SUV values of benign and malignant lesions was statistically significant (*P* < 0.001). The sensitivity, specificity, positive predictive value, negative predictive value, and accuracy of FDG-PET in detecting MPNSTs were 95%, 72%, 71%, 95%, and 82%, respectively. In 5 patients, FDG-PET provided additional information about nontarget lesions that influenced treatment planning. PET and PET/CT were equally sensitive in the detection of MPNST; however, PET/CT proved to be useful in biopsy planning. The authors suggested that FDG-PET and PET/CT are highly sensitive and noninvasive techniques for detecting MPNST in patients with NF1. PET could improve preoperative tumor staging in patients with NF1 and is also able to guide biopsy and direct appropriate therapy in positive cases obviating repetitive surgery and biopsy in negative cases [14].

In 2008, Ferner et al. [15] analyzed the role of FDG-PET and PET/CT for MPNST diagnosis and assessed the clinical indicators of malignancy in 105 NF1 patients with symptomatic PNF. PET scans were carried out 60–90 minutes after FDG injection and delayed local views of the tumor were carried out 240 minutes following FDG injection. Images were evaluated both qualitatively by visual inspection and semiquantitatively by calculation of the maximum SUV measured at the time of the 4-hour scan in order to assess possible malignant change. Excision/biopsy verified the diagnosis and the tumor grading when possible and clinical followup (>2 years) was undertaken in all patients. One hundred and sixteen new lesions were detected including 80 PNF, 5 atypical neurofibromas, 29 MPNSTs, and 2 other cancers. Qualitative assessment of FDG-PET revealed 4 false positive and 3 false negative results. Sensitivity of FDG-PET in diagnosing NF1-associated MPNST was 89% and the specificity was 95%. However, the 3 patients with false negative scans were all low-grade MPNSTs and the sensitivity for high-grade MPNSTs was 100%. SUV was significantly greater in MPNST compared with PNF (*P* < 0.001): mean SUV for 74 PNF was 1.5 ± 1.06; mean SUV for 26 MPNST was 5.7 ± 2.6. No malignant tumors were detected with maximum SUV <2.5 and there were 3 benign tumors with SUV >3.5. However, there was an overlap between benign PNF and MPNST regarding SUV from 2.5 to 3.5. Statistical comparison of SUV between high-grade and low-grade MPNST was significant (*P* = 0.055) but there was considerable overlap between the groups. These results suggested that FDG-PET and PET/CT are sensitive and specific diagnostic tools for NF1-associated MPNST and may guide the biopsy on the area of maximum FDG uptake reflecting the area with the highest grade of the tumor. Considering the overlap between benign PNF and MPNST in SUV range from 2.5 to 3.5, the authors suggested that symptomatic neurofibromas with SUV 3.5 and above should be excised and lesions with SUV between 2.5 and 3.5 should be reviewed clinically. Furthermore, FDG seemed unable to predict tumor grade of MPNST and in discriminating between benign PNF, atypical neurofibromas, and low-grade MPNST [15].

In 2009, Karabatsou et al. [17] evaluated the role of FDG-PET/CT in differentiating benign neurofibroma from MPNST associated with NF1 and in defining regions of suspected PNF where malignant transformation may have started. Nine patients with NF1 who were clinically suspected to have transformation of a PNF to a MPNST were preoperatively evaluated by FDG-PET/CT and MR. PET/CT images were evaluated by visual inspection and calculation of the average and maximal SUV. Biopsies of the tumor were performed on all suspected MPNSTs. Based on histopathology, among the 9 tumors, 2 were clearly benign neurofibromas with homogeneous FDG uptake and a maximal SUV of less than 2.0. Two additional tumors, classified as “cellular neurofibromas,” showed a maximal SUV modestly increased (less than 4.0). The remaining 5 tumors were classified as MPNSTs: 1 tumor grade I, 1 grade III, and 3 grade IV, based on the WHO classification. The maximal SUV was increased at 7.0 or higher, with higher SUV (>7.9) in grades III and IV MPNSTs. All 9 lesions showed heterogeneous MR enhancement. Stratification of the maximal SUV correlated to the proliferative index (Ki-67) and grade of MPNST. A maximum SUV of more than 7.0 was closely correlated to a focus of malignant transformation. In addition, 2 of the 3 grade IV MPNST had already reached metastatic disease at the time of presentation, which was identified by FDG-PET/CT. These data, although on a limited number of cases, demonstrated the potential utility of FDG-PET/CT in improving the diagnosis of MPNST and identifying disseminated disease. A maximum SUV value >4 could be used to differentiate between symptomatic benign PNF and MPNST. Moreover, the addition of CT anatomic imaging to FDG-PET could facilitate targeting biopsies to hypermetabolic areas, to further increase the diagnostic sensitivity [17].

In the same year, Warbey et al. [18] evaluated the use of FDG-PET/CT in patients with symptomatic neurofibromas, in order to revalidate current cut-off values for identification of malignant change, to clarify the value of early and delayed imaging and to examine the relationship between SUV and tumor grade. Sixty-two patients with symptomatic neurofibromas underwent FDG-PET/CT at 90 (early scan) and 240 minutes (delayed scan) after FDG injection. Qualitatively and semiquantitative analyses using the maximum SUV measured on both early and delayed scans were performed. Tumors with a SUV ≥3.5 on delayed imaging were classified as malignant on the basis of PET/CT and those with a SUV <3.5 as benign. The SUV was correlated with histology and with tumor grade. Eighty-five lesions were identified in 62 patients. Excision/biopsy was performed on 39 of the lesions including 8 neurofibromas, 10 atypical neurofibromas, and 21 MPNSTs (11 low-grade and 10 high-grade tumors). Patients with benign neurofibromas were monitored clinically for 2–41 months. On the basis of semiquantitative analysis, 42 tumors were categorized as benign and 43 as malignant. Histological correlation identified 1 false negative scan and 6 false positive scans. Sensitivity of FDG-PET/CT in diagnosing NF1-associated MPNST was 97% (95% CI: 81–99), and the specificity was 87% (95% CI: 74–95). The mean SUV on early imaging was 2.0 for tumors designated as benign and 7.0 for tumors designated as malignant on PET/CT scan. On delayed imaging, the mean SUV was 1.9 and 8.1 for tumors designated as benign and malignant, respectively, on the basis of PET/CT. There was a significant difference in maximum SUV between early and delayed imaging (type irrelevant; *P* = 0.0022). There was a significant difference in SUV also between tumors identified as benign and malignant on PET/CT (time irrelevant, *P* < 0.0001) and between maximum SUV on early and delayed imaging for tumors classified as malignant (*P* = 0.0005) but not for tumors classified as benign on PET/CT (*P* = 0.2). In the tumors with histological correlation, the mean SUV on early imaging were 5.1, 7.3, and 12.0 for atypical neurofibromas, and low- and high-grade MPNSTs, respectively. On delayed imaging, no malignant tumor was identified with SUV <3.2 and there were six benign tumors with SUV >3.5. Mean SUV on delayed imaging for atypical neurofibromas, and low- and high-grade MPNSTs were 5.6, 7.8, and 13.7, respectively. There was a significant difference in SUV between tumor types (time irrelevant, *P* = 0.002); there was also a significant difference in SUV between tumor grades (time irrelevant, *P* = 0.002). A cutoff of SUV of 3.1 on delayed imaging achieved maximal sensitivity (100%) with a specificity of 76.6%; to achieve maximal sensitivity on early imaging a cutoff of SUV of 2.35 would be required, resulting in a specificity of 60%. These results confirmed that FDG-PET/CT is a highly sensitive and specific imaging modality for the diagnosis of MPNST in NF1 patients; furthermore, performing early (90 min) and delayed imaging (at 4 h) and using a cutoff of maximum SUV of 3.5 on delayed imaging allow an accurate lesion characterization with maximal sensitivity. The authors suggested this approach: symptomatic neurofibromas with maximum SUV of 3.5 and above should be excised, lesions with maximum SUV of 2.5–3.5 should be reviewed clinically, and those with an SUV <2.5 considered as benign. Considering the correlation between mean maximum SUV and tumor grade, FDG-PET/CT could be used to grade the malignancy of the tumor [18].

In 2010, Benz et al. [19] also evaluated the ability of FDG-PET/CT to distinguish MPNST from benign peripheral nerve sheath tumors (PNSTs), such as schwannoma and neurofibroma, and assessed whether sporadic and hereditary MPNSTs, schwannoma, and neurofibroma exhibit different glucose metabolic phenotypes. The study population consisted of 34 patients with 40 PNSTs who underwent a presurgical FDG-PET/CT scan. All tumors were characterized histologically, by maximum SUV, and by CT size (tumor maximal diameter). There were 17 MPNSTs (16 high grade and 1 intermediate grade) and 23 benign PNSTs (9 neurofibromas and 14 schwannomas). Twelve MPNSTs were classified as sporadic disease, and 5 MPNSTs developed in patients with NF1. MPNSTs were significantly larger than the benign variants (mean: 7.4 ± 4.1 cm versus 4.8 ± 2.7 cm; *P* = 0.008); however, the range in size of the benign and malignant tumors showed considerable overlap. A ROC analysis revealed that CT tumor size measurements could not reliably distinguish between malignant and benign PNSTs. The mean SUV was significantly higher in MPNST compared with benign PNST (12.0 ± 7.1 versus 3.4 ± 1.8; *P* < 0.001). By ROC curve analysis, maximum SUV reliably differentiated between benign and malignant PNST (area under the ROC curve of 0.97). Interestingly, the difference between MPNST and schwannomas was less prominent than that between MPNST and neurofibromas. A maximum SUV cutoff point ≥6.1 separated MPNST from benign PSNT with a sensitivity of 94% and a specificity of 91% (*P* < 0.001). Sporadic and NF1-associated MPNST showed comparable FDG uptake. These findings demonstrated that semiquantitative FDG-PET analysis can differentiate MPNSTs from neurofibromas with high accuracy and confirmed that size criteria, by CT imaging, cannot make this distinction; in contrast, MPNSTs and schwannomas were less reliably distinguished [19].

#### 3.2.2. Value of FDG-PET and PET/CT in Prediction of PNF Progression to MPNST

In their prospective study, Fisher et al. [16] hypothesized that PNFs with high FDG uptake are more likely to progress in the following year. Eighteen patients with NF1, all clinically stable but considered “high-risk for progression” based upon anatomic location of PNF, were enrolled. MR was performed soon after enrollment and one year following the baseline examination. Percent change in PNF volume from baseline to follow-up scan was calculated. FDG-PET scans were performed within 2 weeks of the baseline MR study. The maximum SUV was calculated for all focally active index lesions and analyzed for correlation with percent change in PNF volume detected in quantitative MRI. Fifteen of 18 patients (83%) showed various degrees of FDG uptake as focal abnormalities. The location of FDG-PET abnormalities in these patients corresponded to that noted on the MR scans. Thirteen cases and 19 lesions were evaluable for PNF volume change at one year. SUV ranged from 0.9 to 4.0 (mean 1.67) in the index lesions. There was a moderate direct correlation between SUV and change in PN volume over the subsequent year (*P* = 0.083). There was a significant difference in the percent increase in PNF volume in the following year for lesions that had an SUV >2 (*n* = 4, median percent change = 27%) compared to those with lower values (*n* = 15, median percent change = 4%) (*P* = 0.016). These findings supported the hypothesis that, since conventional radiographic techniques have limited prognostic value, FDG-PET may be an important imaging modality in patients with NF1 to differentiate benign PNFs which are aggressive and will progress from those that will stay stable. Predicting the rate of growth of these tumors could assist clinician decision making with regard to treatment of PNF and facilitate early intervention in tumors with a high probability of progression [16].

#### 3.2.3. Role of FDG-PET and PET/CT in Prediction of Outcome in Patients with MPNST

Brenner et al. [12] retrospectively assessed the potential of FDG-PET for risk assessment in patients with NF1 and MPNST and evaluated the role of SUV as a parameter for prediction of patient outcome. FDG-PET was performed in 16 patients with NF1 and MPNST. SUV was calculated for each tumor and correlated to tumor grade, as established by the final reports of surgical histopathology, and to patient outcome in terms of survival or death with a mean follow-up period of 20 months since the first PET study (range 4–62 months in all patients). Tumor SUV ranged from 2.1 to 11.6 in the 16 MPNST patients, with a mean of 5.7 ± 2.9. SUV did not show significant differences between grade II (4.7 ± 3.1; range: 2.1–11.0; *n* = 7) and grade III tumors (6.4 ± 2.6; range 3.2–11.6; *n* = 9; *P* = 0.270). Significant differences in SUV, however, were found between patients who were still alive after 36 months (SUV 2.5 ± 0.4; range 2.1–2.8, *n* = 3) and patients who died (6.3 ± 2.7; range 3.2–11.6; *n* = 12, *P* < 0.001). Three patients with tumor grade II had an SUV <3. None of these patients developed metastases or died during a followup of 41–62 months. Thirteen patients with tumor grades II and III had an SUV >3. Only one of these patients was still alive after 20 months; the remaining 12 died within 4–33 months. Based on SUV findings in patients with long-term survival, an SUV of 3.0 as the cutoff identified patients with a favourable prognosis. Using this cutoff, SUV predicted long-term survival with sensitivity, specificity, positive and negative predictive values, and accuracy of 75%, 100%, 100%, 92%, and 94%, respectively. In Kaplan-Meier survival analysis, patients with an SUV >3 had a significantly shorter mean survival time, 13 months, than patients with an SUV <3, in whom the mean survival time was 52 months (*P* = 0.007). Any combination of SUV and tumor histological grade did not improve risk assessment. Tumor grading did not reveal differences in survival time between grades (15 versus 12 months; *P* = 0.141). Therefore, the authors concluded that tumor SUV obtained by FDG-PET is a significant parameter for prediction of survival in NF1 patients with MPNST. FDG-PET as a new risk stratification tool could be useful for the management of NF1 patients with MPNST helping to individualize follow-up regimens [12].

#### 3.2.4. Role of FDG-PET and PET/CT for Surveillance and Management in Pediatric Patients with NF1

In 2005, Wegner et al. [11] reviewed the impact of FDG-PET on the management of pediatric oncology patients over a 10-year period. Diagnoses included 13 PNFs with suspected malignant change and other 152 tumors. A standardised questionnaire was sent to the referring clinicians to determine whether the PET scan had altered management and whether overall the PET scan was thought to be helpful. Taking into account only the 13 patients with PNF, a total of 16 scans were performed and in all cases PET scan was requested for the assessment of possible transformation of a painful, growing PNF to a MPNST. PET findings were confirmed in 12/16 (75%) cases by histology or by clinical followup. PET had led to a change of management in 9/16 (56%) cases. The most frequent change of management was that from biopsy or surgery to no further treatment. PET was considered helpful in 11/16 (69%) cases. Indeed, in seven cases PET saved the child from having an unnecessary invasive procedure. In other cases, PET was helpful by confirming the suspicion of malignant transformation and guiding the biopsy to the most metabolically active area or by changing the surgical approach. The authors concluded that PET could modify the clinical management in pediatric patients with PNF [11].

More recently, Moharir et al. [20] retrospectively analysed the utility of FDG-PET/CT for tumor surveillance in 18 children with NF1, with the specific aims to determine its potential role in the identification of optic pathway gliomas (OPGs) that are likely to progress and in the identification of malignant change in preexisting PNF. FDG avidity of tumors was semiquantitatively analysed by calculating the maximum SUV and was graded into 3 categories: grade 1 or low (SUV <3), grade 2 or intermediate (SUV <4 and >3), grade 3 or intense (SUV >4). There were 7 children with OPG, 7 with PNF, and 4 with both OPG and PNF. A total of 16 PNFs were imaged in 11 patients. Twelve PNFs were grade 1, two were grade 2 and two were grade 3. The SUV ranged from 0.76 to 6.46 (mean: 2.35). Four PNFs demonstrated a SUV >3. Of these, two with grade 2 were mildly symptomatic and have been followed with serial imaging and no biopsies have been performed. The other two patients with grade 3 PNF were symptomatic and in these patients biopsies were performed: both were MPNSTs on tissue diagnosis. PET/CT diagnosed malignant transformation with a sensitivity of 100%, specificity of 85.7%, positive predictive value of 50%, and negative predictive value of 100%. Nineteen OPG, were imaged with PET/CT, and SUV was measured in 16. Ten was grade 1 and three each were grade 2 and grade 3. FDG-avidity reduced from grade 3 to grade 1 in two symptomatic OPGs following chemotherapy, and this was associated with clinical improvement. PET/CT diagnosed symptomatic OPG with a sensitivity of 62.5% and a specificity of 87.5%. From these findings, the authors concluded that, like in adults, clinical symptoms are not reliable indicator of malignant change in PNF. FDG-PET appears to be useful in the diagnosis of malignant change in PNF in children with NF1. Although these data do not support the use of PET screening in the majority of patients with NF1, it is a useful adjunct to MR when malignant change needs to be excluded. FDG-PET/CT may also provide useful information to the surveillance of OPG in childhood NF1—particularly to identify progressive, symptomatic tumors [20].

## 4. Conclusions and General Remarks

From this systematic review of the literature about the role of FDG-PET and PET/CT in patients with NF1, we summarize that the following.

1. FDG-PET and PET/CT are useful and highly sensitive noninvasive methods to identify malignant change in neurogenic tumors in patients with NF1.
2. FDG-PET and PET/CT allow discrimination of MPNSTs from benign neurogenic lesions in NF1; nevertheless, an overlap between benign PNF and MPNST regarding SUV should be considered, resulting in false positive result and a lower specificity. Performing early and delayed imaging (at 4 h) and using a cutoff of maximum SUV were suggested to overcome these possible pitfalls and to obtain an accurate lesion characterization with maximal sensitivity. The ideal SUV cut-off value with the highest sensitivity and specificity is matter of debate because it depends on several factors such as time of measurement after FDG injection, maximum or mean SUV calculation, examination performed with PET or PET/CT scanner, wide variability of SUV values in MPNSTs. Most authors recommend to choose a lower SUV threshold (ranging around 3.5), accepting the risk of some false positive results, but avoiding the risk of failing the early detection of a MPNST. Lesions with low SUV could undergo clinical/radiological followup; lesions with an intermediate SUV could undergo open biopsy or re-evaluation; lesions suspected to be malignant could undergo guided biopsy or resection. The usefulness of FDG-PET and PET/CT in screening symptomatic and asymptomatic neurogenic soft tissue lesions in NF1 patients should be validated through larger multicenter trials.
3. FDG-PET and PET/CT may improve preoperative tumor staging, guide the biopsy, and influence treatment, reducing the number of surgical procedures for benign neurogenic lesions or suggesting early intervention in tumors with a high probability of progression in NF1 patients.
4. Considering the correlation between maximum SUV and tumor grade, FDG-PET could be used to grade the malignancy of the tumor, but there is not agreement in the literature about this topic.
5. Tumor SUV obtained by FDG-PET seems to be a significant parameter for prediction of survival in NF1 patients with MPNST and it seems to allow the identification of MPNST patients with long-term survival independently of histopathological findings. Nevertheless, this conclusion is based on a small sample size and further studies are needed to confirm these preliminary findings.
6. Finally, as in adults, FDG-PET and PET/CT seem to be useful diagnostic tools in children with NF1 for PNF surveillance and clinical management, but larger prospective studies addressing and refining the usefulness and the indications of FDG-PET and PET/CT in this patient population are warranted.

## Figures and Tables

**Table 1:** Characteristics of the included articles and patients.

Authors	Journal	Year	Country	PET device	Number of patients with neurogenic tumors who performed PET (in brackets patients with NF1)	Sex (% male)	Mean age (years)	Number of neurogenic tumor lesions evaluated
Ferner et al. [9]	J Neurol Neurosurg Psychiatry	2000	UK	PET	18 (18)	44%	28	23
Cardona et al. [10]	Eur J Surg Oncol	2003	Germany	PET	13 (5)	38%	46	25
Wegner et al. [11]	Eur J Nucl Med Mol Imaging	2005	UK	PET	13 (n.a.)	46%	15	n.a.
Brenner et al. [12]	Eur J Nucl Med Mol Imaging	2006	Germany	PET	16 (16)	44%	32	16
Bensaid et al. [13]	Ann Dermatol Venereol	2007	France	PET	38 (38)	n.a.	n.a.	49
Bredella et al. [14]	AJR Am J Roentgenol	2007	USA	PET and PET/CT	45 (45)	49%	37	50
Ferner et al. [15]	Ann Oncol	2008	UK	PET and PET/CT	105 (105)	48%	31	114
Fisher et al. [16]	J Neurooncol	2008	USA	PET	18 (18)	50%	14	19
Karabatsou et al. [17]	Neurosurgery	2009	Canada	PET/CT	9 (9)	55%	38	9
Warbey et al. [18]	Eur J Nucl Med Mol Imaging	2009	UK	PET/CT	62 (62)	50%	31	85
Benz et al. [19]	Cancer	2010	Germany/USA	PET/CT	34 (5)	59%	46	40
Moharir et al. [20]	Eur J Nucl Med Mol Imaging	2010	Canada/Australia	PET/CT	18 (18)	44%	8	35

n.a.: not applicable.

**Table 2:** Characteristics of the PET studies.

Authors	Indication for exam	Diagnostic criteria			Number of neurogenic lesions evaluated	MPNST diagnosis	
		Qualitative analysis	Semiquantitative analysis/SUV cutoff	Calculated SUV	(in brackets NF 1 lesions)	Se	Sp
Ferner et al. [9]	Symptomatic NF	Uptake > liver = malignant	—	Mean SUV	23 (23)	100%	87%
Cardona et al. [10]	Suspicious MPNST	Hypermetabolic area	1.8	Median SUV	25 (15)	100%^∗^	83%^∗^
Wegner et al. [11]	Symptomatic NF	—	—	—	13 ( n.a.)	—	—
Brenner et al. [12]	Prediction of MPNST outcome	—	3	Mean SUV	16 (16)	75%	100%
Bensaid et al. [13]	Symptomatic NF	—	Tumour/liver ratio >1.5		49 (49)	100%	86%
Bredella et al. [14]	Symptomatic NF	Uptake > liver = malignant	—	Maximum SUV	47 (47)	95%	72%
Ferner et al. [15]	Symptomatic NF	Uptake > liver and not reduced at 4 hours = malignant	—	Maximum SUV	114 (114)	89%	95%
Fisher et al. [16]	High-risk progression NF	5 point visual scale	2	Maximum SUV	19 (19)	—	—
Karabatsou et al. [17]	Suspicious MPNST	not specified	7	Average and maximal SUV	9 (9)	—	—
Warbey et al. [18]	Symptomatic NF	not specified	3.5 (at 4 hours)	Maximum SUV	85 (85)	97%	87%
Benz et al. [19]	Presurgical evaluation of PNST	—	6.1	Maximum SUV	26 (5)	94%	91%
Moharir et al. [20]	Symptomatic NF	—	>3	Maximum SUV	16 (16)	100%	85%

NF: neurofibromas; MPNSTs: malignant peripheral nerve sheath tumors; PNSTs: peripheral nerve sheath tumors; Se: sensitivity; Sp: specificity; ^∗^: calculated for all 25 neurogenic tumours (15 tumours were detected in 5 NF1 patients) with semiquantitative analysis.
